# Alcohol Use and Misuse Among Chinese Psychiatrists During the Early COVID-19 Pandemic

**DOI:** 10.3389/fpsyt.2022.933814

**Published:** 2022-06-22

**Authors:** Daming Mo, Kaiyuan Min, Rachel Gluck, Feng Jiang, Rui Tao, Feng Geng, Lei Xia, Tingfang Liu, Yuanli Liu, Huanzhong Liu, Yi-lang Tang

**Affiliations:** ^1^Department of Psychiatry, Chao Hu Hospital of Anhui Medical University, Hefei, China; ^2^Department of Psychiatry, School of Mental Health and Psychological Sciences, Anhui Medical University, Hefei, China; ^3^Department of Psychiatry, Hefei Fourth People's Hospital, Hefei, China; ^4^State Key Laboratory of Medical Molecular Biology, Institute of Basic Medical Sciences, Chinese Academy of Medical Sciences and Peking Union Medical College, Beijing, China; ^5^Department of Psychiatry and Behavioral Sciences, Emory University, Atlanta, GA, United States; ^6^School of International and Public Affairs, Shanghai Jiao Tong University, Shanghai, China; ^7^Institute of Healthy Yangtze River Delta, Shanghai Jiao Tong University, Shanghai, China; ^8^Department of Psychiatry, Second Affiliated Hospital of Anhui Medical University, Hefei, China; ^9^School of Health Policy and Management, Chinese Academy of Medical Sciences and Peking Union Medical College, Beijing, China; ^10^Atlanta Veterans Affairs Medical Center, Decatur, GA, United States

**Keywords:** alcohol use, alcohol misuse, psychiatrists, COVID-19 pandemic, alcohol use disorder

## Abstract

**Aim:**

Survey alcohol use and misuse among Chinese psychiatrists during the Coronavirus diseases 2019 (COVID-19) pandemic.

**Methods:**

We conducted a large-scale, nationwide online survey of psychiatrists regarding their alcohol use during the pandemic. The Alcohol Use Disorder Identification Test-Concise (AUDIT-C) was used to assess alcohol use and misuse.

**Results:**

Of 3,815 psychiatrists who completed the survey, alcohol use and misus were 47.5% and 8.2%, respectively, and both were significantly higher in males. The majority (59%) reported no change in alcohol use during the pandemic, one-third (34.5%) reported a decrease, and 6.5% reported an increase. Alcohol misuse was associated with middle-age (OR = 1.418), male sex (OR = 5.089), Northeast China (OR = 1.507), cigarette-smoking (OR = 2.335), insomnia (OR = 1.660), and regular exercise (OR = 1.488). A master's degree (OR = 0.714) and confidence in clinical work (OR = 0.610) were associated with less alcohol misuse. Those who reported a decrease in alcohol use during the pandemic were more likely to be male (OR = 2.011), located in Northeast China (OR = 1.994), and feel confident in their clinical work (OR = 1.624). Increased alcohol use was significantly associated with insomnia (OR = 3.139).

**Conclusions:**

During the COVID-19 pandemic, alcohol use and misuse among Chinese psychiatrists declined. While males were more likely to misuse alcohol, they were also more likely to have reduced their intake. Age, location, and lifestyle factors also predicted alcohol use and misuse. Further examination of specific factors that reduced alcohol use and misuse may help guide public health efforts to sustain the lower rates beyond the pandemic.

## Introduction

Alcohol use disorder (AUD) is prevalent in the general population and is estimated at 3.4% in Europe (1). Recent reports indicate that China's per capita alcohol consumption increased by 80% between 2005 and 2018 (2), faster than anywhere else in the world (3). Physicians play an essential role in preventing, screening, and managing AUD (4). However, physicians are also at risk of hazardous alcohol consumption, yet data describing physicians' alcohol use are scarce. Recent international studies show alcohol misuse among physicians varies considerably, with 12.6% misuse found in French physicians and 32% in Spanish primary health care (5–7). One self-report survey conducted in 1,418 physicians (1,447 contacted) in east China's Jiangsu province in 2008 showed an alcohol misuse incidence of 14.0% (40.4% for men and 2.6% for women) (8). A 2019 survey of Chinese mental health workers showed an overall alcohol consumption rate of 41.8% (53.9% of psychiatrists, 36.2% of nurses, and 40.5% of clinical psychologists) (9). Alcohol use and misuse were relatively common among mental health professionals in China, which may negatively impact their physical and psychological wellbeing as well as impair patient care. Our previous study recommended interventions to promote healthier use of alcohol in this professional group, especially among at-risk subgroups (9).

The Coronavirus disease 2019 (COVID-19) is a viral respiratory infection that reached pandemic levels in March 2020 (10), immediately following the collection of these data from Chinese mental health workers. COVID-19 had a significant impact on the physical health and mental well-being of the global population. As social distancing and quarantine guidelines came into effect, people worldwide were exposed to many psychosocial stressors, from decreased opportunities to socialize to job loss (11). Healthcare workers in particular were significantly affected by the pandemic, as they were pressed to work longer hours with very sick patients. A systematic review found that healthcare workers were at increased risk of mental health difficulties during the pandemic, including anxiety and depressive symptoms (12–14). Research has shown that many use alcohol or other substances to cope with negative mood states and stress (15, 16), and those with pre-existing AUD tend to increase their alcohol in response to increases in stress (17).

A longitudinal study in China during the SARS outbreak showed that hospital staff used alcohol as a coping method (18). Some respondents who began using alcohol during the SARS pandemic indicated continued use once the pandemic was over (18). Whether or not alcohol consumption has similarly increased during the COVID-19 pandemic has been a topic of debate (19). A growing literature has investigated the effects of the COVID-19 pandemic on alcohol use among different populations, including college students, community adults, and older adults, and the findings are mixed (19–25). Some studies have found that most individuals continued to use alcohol at the same rate as before the COVID-19 pandemic (21–23, 26). Other studies have shown that the general population's alcohol consumption increased significantly (24, 27). By contrast, studies conducted in the general population in China found decreased alcohol consumption during the pandemic (28). Alcohol consumption may be one of the ways people respond to the remarkable changes associated with the COVID-19 pandemic, and understanding alcohol use among psychiatrists is of importance, as their knowledge, perceptions, and attitudes often affect their interactions with patients. Furthermore, investigating alcohol use may shed light on overall mental health and coping behaviors of this professional population.

There are several possible explanations for the differences in study findings including variations in sample makeup, settings, cultures, and the exact timing of the study with respect to the pandemic (such as during the lockdown, acute phase, or chronic phase). In addition to inconsistent findings, little is known about the specific factors associated with changes in alcohol use during the pandemic. Given prior data about alcohol use to cope with stress, changes in use during increases in stress, and evidence of increased use in healthcare workers in China during the prior SARS pandemic, we hypothesized that healthcare workers would be at increased risk of alcohol use and misuse during the COVID-19 pandemic. Therefore, this study's main objective is to explore the changes in alcohol use among Chinese psychiatrists during the COVID-19 pandemic based on a large, nationally representative sample.

## Methods

### Study Design, Setting, and Participants

The study was a large-scale, nationwide online survey of Chinese healthcare professionals conducted in January 2021. In total, 21,858 healthcare professionals (including psychiatrists, psychiatric nurses, and psychotherapists) from 41 major psychiatric tertiary hospitals in 29 provinces in China were approached to participate in the survey. A total of 18,713 people completed the survey, including 3,815 psychiatrists. The survey was conducted anonymously through WeChat, an online social media application widely used in China. To avoid double registration each WeChat account could only submit one questionnaire. We collected socio-demographic data, alcohol use, and information about other health-related behavior (exercise, sleep, and cigarette smoking). The Alcohol Use Disorder Identification Test (AUDIT-C) was used to examine patterns of alcohol use.

### Questionnaire Design

We developed the online questionnaire based on the existing literature and expert opinion. Before the survey was made available to all participants, a pilot study was conducted in a small sample (*N* = 332, including nurses, doctors, and psychologists) to assess the readability of the questions. AUDIT-C is a concise version of the AUDIT, and it has been demonstrated to have similar accuracy to the full AUDIT (29–31). The total AUDIT-C score ranges from 0 to 12, with a higher score indicating more severe alcohol use. The Chinese version of the AUDIT has been validated (32) and used in different settings (9, 33, 34), the details in AUDIT-C scoring can be found elsewhere (9, 33, 34). Using the AUDIT-C scores, participants were categorized as no alcohol users (the total score = 0) and alcohol users (the total score ≥1). The alcohol use group was further divided into low-risk users of alcohol [the total score of <3 (women) and <4 (men)] and probable alcohol misuse (hazardous, harmful, or dependent) [the total score of ≥3 (women) and ≥4 (men)] (25).

Socio-demographic and occupational characteristics such as age (years), sex (male/female), marital status (married, single, divorced, or widowed), region (East, Central, West, or Northeast China), and education (degree) were collected. We referred to the World Health Organization criteria to designate age groups (20–34, 35–49, ≥50 years). The health-related behaviors included physical exercise, sleep, and cigarette smoking. In this study, we defined “regular exercise” as exercising at least three times per week in the past month according to the recommendations of the National Fitness Guideline. “Frequent insomnia” was defined as having sleep disturbances (difficulty falling asleep, difficulty maintaining sleep, or waking up early) at least three times per week in the past month. Participants were considered to have a “smoking habit” if they had a cumulative history of smoking more than 100 cigarettes in their lifetime (35). We also collected information on whether psychiatrists had been on the “frontline” of COVID-19 (treating COVID-19 patients) and their overall attitude and confidence about being a healthcare professional going forward (negative, neutral, positive).

### Statistical Analysis

The sample distribution was conducted by frequency calculated for categorical variables. For the statistical analysis, the one-sample K-S test was utilized to assess the normality of numeric variables. The Chi-square test was used for univariate analysis. Multiple logistic regression was used to calculate OR value and 95% confidence interval, and multivariate analysis was performed. All statistical analyses used IBM. Statistical Package of Social Sciences (SPSS version 22.0) with two-tailed *p* values of 0.05 for statistical significance.

## Results

### Demographic Characteristics of Participants

As shown in Table 1, 60% of the participants were female, more than four-fifths (85.3%) were younger than 50 and 80% were married. We found that 1,811 subjects (47.5%) self-identified as current alcohol users. Significant differences were found between those with and without alcohol use with respect to the demographic (age group, sex, education, marital status, region) and lifestyle (smoking habit, frequent insomnia) factors, and experience of working in the frontline of COVID-19, perceived confidence in clinical work (all *p* < 0.05) (Table 1).

**Table 1:** Basic features of 3,815 psychiatrists, and subgroup comparisons between those endorsed alcohol use, alcohol misuse and those who did not.

**Variables**	**All participants**	**Alcohol use**		**Alcohol misuse**		**Alcohol use**	**Alcohol misuse**
	***n =* 3,815**	**Yes** **(*n =* 1,811)**	**No** **(*n =* 2,004)**	**Yes** **(*n =* 316)**	**No** **(*n =* 3,499)**	**χ^2^ (*(p-value)*)**	**χ^2^ (*(p-value)*)**
Age group (*N*, %)							
20–34	1,390 (36.4)	590 (42.4)	800 (57.6)	79 (5.7)	1,311 (94.3)	23.721 (<0.001)	24.835 (<0.001)
35–49	1,865 (48.9)	926 (49.7)	939 (50.3)	169 (9.1)	1,696 (90.9)		
≥50	560 (14.7)	295 (52.7)	265 (47.3)	68 (12.1)	492 (87.9)		
Sex (*N*, %)							
Male	1,526 (40.0)	1,124 (73.7)	402 (26.3)	253 (16.6)	1,273 (83.4)	699.38 (<0.001)	230.420 (<0.001)
Female	2,289 (60.0)	687 (30.0)	1,602 (70.0)	63 (2.8)	2,226 (97.2)		
Education (*N*, %)							
College or-							
bachelor degree	2,460 (64.5)	1,232 (50.1)	1,228 (49.9)	247 (10.0)	2,213 (90.0)	25.253 (<0.001)	28.163 (<0.001)
Master degree	1,122 (29.4)	462 (41.2)	660 (58.8)	57 (5.1)	1,065 (94.9)		
Doctoral degree	233 (6.1)	117 (50.2)	116 (49.8)	12 (5.2)	221 (94.8)		
Marital status (*N*, %)							
Married	3,042 (79.7)	1,413 (46.4)	1,629 (53.6)	261 (8.6)	2,781 (91.4)	6.783 (0.034)	1.805 (0.406)
Single	629 (16.4)	320 (50.1)	309 (49.9)	44 (7.0)	585 (93.0)		
Divorced or-	149 (3.9)	78 (52.3)	66 (47.7)	11 (7.4)	133 (92.6)		
widowed							
Region (*N*, %)							
East China	1,544 (40.5)	680 (44.0)	864 (56.0)	98 (6.3)	1,446 (93.7)	35.461 (<0.001)	16.058 (0.001)
Central China	820 (21.5)	364 (44.4)	456 (55.6)	76 (9.3)	744 (92.7)		
West China	1,045 (27.4)	528 (50.5)	517 (49.5)	94 (8.9)	951 (91.1)		
Northeast China	406 (10.6)	239 (58.9)	167 (41.1)	48 (11.8)	358 (88.2)		
Smoking habit							
No (%)	3,422 (89.7)	1,483 (43.3)	1,939 (56.7)	209 (6.1)	3,213 (93.9)	227.586 (<0.001)	206.957 (<0.001)
Yes (%)	393 (10.3)	328 (83.5)	65 (16.5)	107 (27.2)	286 (72.8)		
Frequent insomnia							
No (%)	3,167 (83.0)	1,502 (47.4)	1,665 (52.6)	232 (7.3)	2,935 (92.7)	0.014 (0.904)	22.503 (<0.001)
Yes (%)	648 (17.0)	309 (47.7)	339 (52.7)	84 (13.0)	564 (87.0)		
Regular exercise							
No (%)	3,493 (91.6)	1,643 (47.0)	1,850 (53.0)	272 (7.8%)	3,221 (92.2)	3.120 (0.077)	13.407 (<0.001)
Yes (%)	322 (8.4)	168 (52.2)	154 (47.8)	44 (13.7)	278 (86.3)		
Participated in the frontline work of COVID-19							
No (%)	2,923 (76.6)	1,329 (45.5)	1,594 (54.5)	226 (7.7)	2,697 (92.3)	20.124 (<0.001)	5.002 (0.025)
Yes (%)	892 (23.4)	482 (54.0)	410 (46.0)	90 (10.1)	802 (89.9)		
Confidence in medical work^a^							
Neutral (%)	1,093 (29.7)	492 (45.0)	601 (55.0)	90 (8.2)	1,003 (91.8)	4.434 (0.109)	1.588 (0.452)
Negative (%)	2,221 (60.4)	1,085 (48.9)	1,136 (51.1)	195 (8.8)	2,026 (91.2)		
Positive (%)	365 (9.9)	177 (48.5)	1,88 (51.5)	25 (6.8)	340 (93.2)		

*Frequent insomnia: having sleep disturbances at least three times per week; regular exercise: exercising at least three times per week*.

a *n = 3,679*.

### Alcohol Misuse and Related Factors Among Psychiatrists

The overall prevalence of alcohol misuse among the participants sampled was 8.2% during the COVID-19 pandemic and varied significantly between male and female psychiatrists (16.6% prevalence in males and 2.8% in females). Rates of alcohol use increased with age, from 5.1% for low-age (20–34 years group) to 9.1% (35–49 years group) and 12.1% (≥50 years group) for middle and high-age. Compared to those with college/medical degrees (10.0%), those with Master's (5.1%) or doctoral degrees (5.2%) had significantly lower rates of alcohol misuse (*p* < 0.01). The rate of alcohol misuse among psychiatrists in northeast China was significantly higher than that in east regions (11.8 vs. 6.3%, *p* < 0.01). The smoking rate among alcohol misusers was more than 4 times higher than that of non-alcohol misusers (27.2 vs. 6.1%, *p* < 0.01). Alcohol misuse was significantly more common in people with frequent insomnia than those without insomnia (13.0 vs. 7.3%, *p* < 0.01). Those who reported regular exercise had higher rates of alcohol misuse than non-exercisers (13.7 vs. 7.8%, *p* < 0.01). Alcohol misuse was higher among psychiatrists who participated in the frontline work of COVID-19 than those with no such experience (10.1 vs. 7.7%, *p* < 0.01) (Table 1).

### Factors Associated With Alcohol Misuse in a Multiple Logistic Regression

We used AUDIT-C cut-off scores to divide the sample (*N* = 3,815) into two groups, those with probable alcohol misuse (*N* = 316) and those without (*N* = 3,499). We used multiple logistic regression to examine the association between probable alcohol misuse and demographic variables (Table 2). The references of the categorical variables were defined as shown in Table 2. During the COVID-19 pandemic, a higher risk of alcohol misuse was associated with the 35–50 year group (OR = 1.418; 95%CI = 1.012–1.988), male sex (OR = 5.089; 95% CI = 3.719–6.965), working in Northeast China (OR =1.507; 95%CI = 1.011–2.246), smoking habit (OR = 2.335; 95%CI = 1.742–3.131), frequent insomnia (OR = 1.660; 95% CI = 1.244–2.213) and regular exercise (OR = 1.488; 95%CI = 1.022–2.165). An add-on Master's degree and positive confidence in medical work were significantly associated with a lower risk of alcohol misuse among alcohol users (OR = 0.714; 95%CI = 0.514–0.927 and OR = 0.610; 95%CI = 0.377–0.987, respectively).

**Table 2:** Multiple logistic regression examining individual characteristics associated with alcohol misuse in psychiatrists in China.

**Variables**	**OR**	**95% CI**	***P-value***
Age group (ref. 20–34)			
35–50	1.418	1.012–1.988	0.042
≥50	1.269	0.839–1.921	0.259
Sex (ref. Female)			
Male	5.089	3.719–6.965	<0.001
Education (ref. college or Bachelor degree)			
Master degree	0.714	0.514–0.992	0.045
Doctoral degree	0.639	0.331–1.234	0.182
Marital status (ref. married)			
Single	1.176	0.784–1.765	0.434
Divorced or widowed	0.881	0.447–1.738	0.716
Region (ref. East China)			
Central China	1.370	0.972–1.933	0.073
West China	1.227	0.886–1.699	0.219
Northeast China	1.507	1.011–2.246	0.044
Smoking habit (ref. no)			
Yes	2.335	1.742–3.131	<0.001
Frequent insomnia (ref. no)			
Yes	1.660	1.244–2.213	0.001
Regular exercise (ref. no)			
Yes	1.488	1.022–2.165	0.038
Participated in the frontline work of			
COVID-19 (ref. no)			
Yes	1.068	0.808–1.410	0.645
Confidence in medical work (ref. neutral)			
Negative	0.969	0.733–1.281	0.826
Positive	0.610	0.377–0.987	0.044

### Reported Changes in Alcohol Use During the COVID-19

Among all participants, 1,035 (59.0%) reported no change in alcohol use during the COVID-19 pandemic, 605 (34.5%) reported a decrease, and 114 (6.5%) reported an increase. We examined the differences in alcohol use across subgroups by demographic features, health-related behaviors, and direct experience working with COVID-19 patients. With respect to sex effects, significantly more males were classified as either increased or decreased alcohol use than females (39.5 vs. 26.5%; 7.6 vs. 4.8%, both *p* < 0.01). For those who reported a decrease in alcohol use, significantly higher rates were found in those with a college/medical degree (37.5%) than those with an add-on master's degree (27.7%) (*p* < 0.01). Nearly half (46.4%) of those who reported a decrease in alcohol use were working in Northeast China. The rate of psychiatrists reporting an increase in alcohol was significantly higher (14.2%) than those without insomnia (4.9%) (*p* < 0.01). The rate of reported decrease was significantly higher (44.9%) in those who reported less frequent or no exercise, compared to those with regular exercise (33.4%, *p* < 0.01). Those who reported feeling confident in their medical work were more likely to report a decrease in alcohol use (51.4%), compared to both those with neutral (36.6%) or negative attitudes toward their medical work (30.8%) (both *p* < 0.01). There were no significant differences in change in alcohol use during the pandemic concerning age, marital status, frontline experience with COVID-19, or smoking habit (Table 3).

**Table 3:** Difference between the studied variables and alcohol use changing during the COVID-19 among 1,754 psychiatrists in China.

**Variables**	**Alcohol use**			**χ^2^**	***p-value***
	**Decrease** ***n =* 605**	**Unchanged** ***n =* 1,035**	**Increased** ***n =* 114**		
Age group (%)					
20–34	197 (34.4)	345 (60.2)	31 (5.4)	5.926	0.205
35–49	295 (33.0)	533 (59.6)	66 (7.4)		
≥50	113 (39.4)	157 (54.7)	17 (5.9)		
Sex (%)					<0.001
Male	427 (39.4)	573 (53.0)	82 (7.6)	42.817	
Female	178 (26.5)	462 (68.7)	32 (4.8)		
Education (%)					
College or-Bachelor degree	449 (37.5)	664 (55.5)	84 (7.0)	19.625	0.001
Master degree	125 (27.7)	301 (66.8)	25 (5.5)		
Doctoral degree	31 (29.2)	70 (66.1)	5 (4.7)		
Marital status (%)					
Married	484 (35.5)	783 (57.5)	95 (7.0)	6.599	0.161
Single	97 (30.7)	203 (64.2)	16 (5.1)		
Divorced or widowed	24 (31.6)	49(64.5)	3 (3.9)		
Region (%)					
East China	203 (29.9)	434 (63.8)	43 (6.3)	2.5.011	<0.001
Central China	102 (33.2)	181 (59.0)	24 (7.8)		
West China	189 (35.8)	301 (57.0)	38 (7.2)		
Northeast China	111 (46.4)	119 (49.8)	9 (3.8)		
Smoking habit (%)					
No	485 (33.8)	862 (60.1)	87 (6.1)	4.911	0.086
Yes	120 (37.5)	173 (54.1)	27 (8.4)		
Frequent insomnia					
No (%)	505 (34.8)	876 (60.3)	71 (4.9)	36.319	<0.001
Yes (%)	100 (33.1)	159 (52.6)	43 (14.2)		
Regular exercise					
No (%)	530 (33.4)	949 (59.8)	108 (6.8)	9.971	0.007
Yes (%)	75 (44.9)	86 (51.5)	6 (3.6)		
Participated in the frontline work of					
COVID-19					
No (%)	441 (34.7)	753 (59.2)	78 (6.1)	1.032	0.597
Yes (%)	164 (34.0)	282 (58.5)	36 (7.5)		
Confidence in medical work					
Neutral (%)	180 (36.6)	282 (57.3)	30 (6.1)	31.539	<0.001
Negative (%)	334 (30.8)	672 (61.9)	79 (7.3)		
Positive (%)	91 (51.4)	81 (45.8)	5 (2.8)		

### Factors Associated With Changes in Alcohol Use in a Multiple Logistic Regression

We also performed multiple logistic regression analysis to examine the associations between reported changes in alcohol use and other variables. During the COVID-19 pandemic, compared to the unchanged group, those who reported a decrease in alcohol use were more likely to be male (OR = 2.011; CI = 1.585–2.552), working in Northeast China (OR = 1.994; CI = 1.432–2.777), and feeling confident in clinical work (OR = 1.624; CI = 1.141–2.363) (Table 4). On the other hand, compared to those who reported no changes in alcohol use, those who reported an increase in alcohol use were more likely to be male (OR = 1.974; CI = 1.235–3.157), and with reported frequent insomnia (OR = 3.139; CI = 2.043–4.825) (Table 4).

**Table 4:** Multiple logistic regression examining individual characteristics associated with alcohol use changed.

**Variables**	**Drinking less than before^a^**		**Drinking more than before^a^**	
	**OR (95% CI)**	***P***	**OR (95% CI)**	***P***
Age group (ref. 20–34)				
35–50	0.842 (0.542–1.104)	0.213	1.049 (0.620–1.774)	0.859
≥50	0.896 (630–1.275)	0.541	0.740 (0.358–1.528)	0.415
Sex (ref. female)				
Male	2.011 (1.585–2.552)	<0.001	1.974 (1.235–3.157)	0.005
Education (ref. college or Bachelor degree)				
Master degree	0.687 (0.528–0.894)	0.005	0.655 (0.393–1.093)	0.105
Doctoral degree	0.821 (0.514–1.313)	0.441	0.653 (0.244–1.750)	0.397
Marital status (ref. married)				
Single	0.811 (0.587–1.121)	0.204	0.659 (0.342–1.272)	0.214
Divorced or widowed	0.898 (0.534–1.510)	0.685	0.541 (0.159–1.838)	0.324
Region (ref. East China)				
Central China	1.031 (0.756–1.406)	0.847	0.991 (0.562–1.748)	0.974
West China	1.204 (0.915–1.585)	0.185	1.006 (0.602–1.680)	0.982
Northeast China	1.994 (1.432–2.777)	<0.001	0.620 (0.281–1.366)	0.235
Smoking habit (ref. no)				
Yes	0.815 (0.612–1.085)	0.160	1.045 (0.625–1.746)	0.866
Frequent insomnia (ref. no)				
Yes	1.126 (0.847–1.496)	0.415	3.139 (2.043–4.825)	<0.001
Regular exercise (ref. no)				
Yes	1.350 (0.955–1.909)	0.089	0.574 (0.236–1.392)	0.219
Participated in the frontline work of				
COVID-19 (ref. no)				
Yes	0.926 (0.731–1.174)	0.528	1.137 (0.729–1.774)	0.572
Confidence in medical work (ref. neutral)				
Negative	0.784 (0.619–0.992)	0.784	1.016 (0.641–1.612)	0.945
Positive	1.624 (1.141–2.363)	0.008	0.548 (0.201–1.492)	0.239

a *Ref. group reported no change in drinking*.

## Discussion

This was the first study to examine alcohol use and misuse in psychiatrists during the COVID-19 pandemic in China. We found that alcohol use and misuse were relatively common among Chinese psychiatrists, with 47.5% reporting alcohol use and 8.2% for probable alcohol misuse. The survey found that alcohol misuse was significantly associated with younger age, male sex, region (Northeast China), education (Master's degree), cigarette use, frequent insomnia, regular exercise, and working experience on the COVID-19 front lines. Changes in alcohol use during the pandemic were significantly associated with male sex, education (Master's degree), region (Northeast China), frequent insomnia, regular exercise, and perceived confidence in clinical work. Specific risk factors for psychiatrists' alcohol misuse during the pandemic were male sex, working in Northeast China, lower levels of education, cigarette use, and regular exercise. Confidence in one's ability to provide adequate care was a protective factor. Compared with those who reported no change in alcohol use, those who reported an increase in alcohol use were more likely to be male, and/or with confidence in clinical work. In contrast, those who reported increased alcohol use were more likely to have frequent insomnia.

### Alcohol Use/Misuse and Smoking Among Chinese Psychiatrists

In 2019, before the pandemic started, our group conducted a similar national survey among mental health professionals (9) using the same screening tool (AUDIT-C) and cutoffs. The current study included a few additional questions: for example, we asked the participants to rate the change in their alcohol consumption compared to before the pandemic. Compared with the findings of the 2019 study, we found that the rates of both alcohol use and alcohol misuse were significantly lower (9) (47.5 vs. 53.9%, *p* < 0.01; 8.2 vs. 10.2%, *p* < 0.01). Among all participants, the majority (59%) reported no change in alcohol use during the pandemic, but more than one-third (34%) reported a decrease in alcohol use, and only 6.5% reported an increase. These findings are overall consistent with the results from another study in China, which surveyed 2,229 participants who were predominantly employed (>90%) and college-educated (>85%), which also showed alcohol use slightly decreased during COVID-19 compared with before (28). However, our findings are different from most studies during the pandemic era from other parts of the world. A meta-analysis of 45 articles on alcohol and other substance use during the COVID-19 pandemic showed an overall increase in alcohol consumption compared to pre-pandemic drinking levels (36).

The unique findings of our study are intriguing. While there are several possible explanations for the overall decrease in alcohol use in this population, two plausible explanations are related to the Chinese government's response to the epidemic and cultural practices in China. For example, the lockdown and social restrictions imposed by the Chinese government were probably among the most-strict worldwide. One report indicated a 90% reduction in open restaurants during the pandemic (37). Social gatherings, including drinking in social settings, were also dramatically reduced and were almost non-existent (37). In China, drinking in the social environment plays a significant role in alcohol use and misuse (3), especially among those with higher socioeconomic status, such as physicians. Before the pandemic, physicians often attended work-related social dinners where alcohol intake was culturally expected. The restrictions during the pandemic likely contributed to the overall alcohol use decrease by reducing social gatherings where alcohol would be used as well as the gifting of alcohol to physicians in thanks. Similar findings were reported in Spain during the pandemic. Rodrigues et al. interviewed 179 Spanish and 179 British participants about drinking behavior before and during the COVID-19 lockdown. They found interesting cultural differences: during the lockdown, Spanish participants reported a decrease in alcohol use while British participants reported no change in their consumption habits. The authors suggested that the decrease in alcohol consumption in Spanish participants may be related to the absence of a social context that culturally condones alcohol use, while British culture may allow for alcohol use outside of social gatherings (38).

While “social” opportunities for alcohol use decreased during COVID-19, another study suggested emotional coping motivated those who increased their alcohol use (39). In fact, coping motives were the strongest predictors of increased alcohol use during the lockdown (40). During the pandemic, healthcare workers may have more medical knowledge and resources to cope with stress, and they may demonstrate a lower burden of mental distress than the general population (41).

Compared with the 2019 study, we also observed a decline in cigarette smoking among psychiatrists (9). Similar to the findings of the 2019 study, we again confirmed that cigarette smoking was significantly associated with alcohol misuse (OR = 2.3). The link between alcohol use and cigarette smoking has been well-documented (42). Our finding of the association between smoking and alcohol misuse among psychiatrists was consistent with other studies conducted in China (43) and other countries (44).

### Demographics and Alcohol Misuse

We found that the rates of alcohol use and misuse were significantly higher in males than females, and male sex was a significant risk factor for alcohol misuse. This gender association has been repeatedly reported in different populations and different cultures (45–47), but the sex gap in our sample is much greater than in most studies in western countries (47). Many studies showed that male doctors had a higher rate of alcohol misuse (48–50). A recent survey of healthcare professionals in a hospital in China also found that male medical workers reported a strikingly high rate of alcohol use than their female colleagues (40.4% for males and 2.6% for females) (8). Gender differences were expressed in different patterns of alcohol consumption and related behaviors, including alcohol use disorders (51). The factors contributing to the differences are multiple and often involve biological (e.g., neurotransmitters, hormones, genetics), psychological (e.g., personality traits, coping mechanisms), and social (role expectation, cultural norms, etc.) factors (47). The traditional Chinese culture and gender stereotyping likely contribute to the striking gender differences in alcohol (and cigarette) use, as Chinese culture is generally disapproving of and often discriminatory against women who drink or smoke.

We found that the middle age group (35–49 years group) was a risk factor for alcohol misuse, consistent with another recent study (9). Middle age is in the career development period who have frequent social activities and experience higher social and career pressures. Since it usually takes 5–10 years or longer to develop alcohol dependence (52), attention needs to be directed to the younger group to provide early intervention.

The 2019 survey from our group found that being divorced or widowed was significantly associated with alcohol misuse (9). The current study also found that the rate of alcohol use among divorced or widowed people was significantly higher than that of married people, however, we did not find a significant association between marital status and alcohol misuse. The previous hypothesis was that marriage's psychological and social aspects, particularly health-monitoring spousal interactions, may protect against the development of alcohol use disorder (53). During the COVID-19 pandemic, many single and divorced individuals returned to their families of origin, which may have decreased their drinking behavior.

Consistent with prior studies, our survey found that an add-on master's degree was a protective factor for alcohol misuse. A recent study of 329 people ages 18–65 in China who consume alcohol found that those with lower education levels drink more heavily than those with higher education levels (54). However, patterns of alcohol use across a lifetime may vary by education as well, as one study in Denmark involving 7,019 men seeking outpatient AUD treatment showed that patients with higher educational levels were likely to have a later onset and treatment of AUD (55).

### Lifestyle and Alcohol Misuse

We found that alcohol misuse was associated with lifestyle behaviors such as exercise and insomnia. Specifically, those with frequent insomnia were more likely to report increased alcohol use during the pandemic. One meta-analysis showed that healthcare professionals fighting COVID-19 are often affected by psychiatric disorders such as depression, anxiety, distress, and insomnia (13). Alcohol is often a coping mechanism to deal with negative mood states and insomnia (17, 56). A study of frontline health workers in Australia during the pandemic found that over a quarter of healthcare workers reported increased alcohol use associated with a history of poor mental health and worse personal relationships (57). In the current study, we found that Chinese psychiatrists' alcohol use and misuse have declined, and more than 93%of them reported no change or decrease in alcohol use. Perceived confidence in clinical work was a protective factor for alcohol misuse, and working on the frontline of COVID-19 did not seem to contribute significantly to alcohol use or misuse.

### Limitations

Some limitations of this study need to be acknowledged. First, we used a screening instrument to assess alcohol use, and we did not have data on the actual diagnosis. The cut-off scores for “alcohol misuse” were based on other studies. Therefore, the actual rates need to be taken with some caution. Second, we only targeted psychiatrists in the top-tier psychiatric hospitals in the capital cities of each province, and we did not include hospitals in smaller towns or rural areas. Third, as most data, such as alcohol use, and the change in alcohol use during the pandemic, were merely based on self-report, this may introduce bias into the findings with participants intentionally or unintentionally underestimating their consumption. Furthermore, as with all online surveys, self-selection bias cannot be ruled out. Fourth, the time frames for different variables were different: it was the past year for alcohol, a lifetime for smoking, and the last month for exercise and insomnia, and this may affect our findings. Fifth, the severity of the pandemic and the restrictiveness of infection prevention standards in different regions were different. The situations were often rapidly evolving, confounding the drinking behavior. Furthermore, we did not data on the individual experience of, and exposure to the COVID-19 and the associated restrictive measures, which were likely to affect some of the findings. Finally, other studies referenced in the above comparisons may have used different instruments or definitions (cut-off scores), thus limiting the true comparability.

## Conclusion

Contrary to our prediction, alcohol use and misuse among Chinese psychiatrists actually decreased during the COVID-19 pandemic. Though males were more likely to misuse alcohol, they were also more likely to have reduced their intake during the pandemic. Psychiatrists who were younger, practiced in Northeast China, smoked cigarettes, and/or experience frequent insomnia were also more likely to misuse alcohol. This survey identified essential factors associated with alcohol use and misuse, which may guide the development of alcohol misuse detection and intervention practices for this professional group. However, more research is needed to explore specific factors underlying drinking behavior among mental health professionals, both during and after the pandemic.

## Data Availability Statement

The raw data supporting the conclusions of this article will be made available by the authors, without undue reservation.

## Ethics Statement

Before this study was initiated, ethical approval was obtained from the Ethical Committee (IRB) at the Chao hu Hospital of Anhui Medical University (202002-kyxm-02). The survey was anonymous. Participants' consent was obtained when they accessed the online questionnaire.

## Author Contributions

J, HL, and Y-lT: study design. DM, TL, YL, KM, RT, FG, LX, and Y-lT: collection, analyses, and interpretation of data. DM: drafting the first version of the manuscript. RG and Y-lT: critical revision of the manuscript. All authors: approval of the final version for publication.

## Funding

This study was supported by the National Clinical Key Specialty Project Foundation (CN) and the Beijing Medical and Health Foundation (Grant No. MH180924).

## Conflict of Interest

The authors declare that the research was conducted in the absence of any commercial or financial relationships that could be construed as a potential conflict of interest.

## Publisher's Note

All claims expressed in this article are solely those of the authors and do not necessarily represent those of their affiliated organizations, or those of the publisher, the editors and the reviewers. Any product that may be evaluated in this article, or claim that may be made by its manufacturer, is not guaranteed or endorsed by the publisher.
